# Evaluation of Norwegian cancer hospitals' Web sites and explorative survey among cancer patients on their use of the Internet

**DOI:** 10.2196/jmir.3.4.e30

**Published:** 2001-12-26

**Authors:** Jan Norum

**Affiliations:** ^1^Norwegian Centre for TelemedicineUniversity Hospital of TromsøNorway

**Keywords:** Internet, information, consumer information, Web site, World Wide Web, cancer care facilities, cancer hospitals, hospitals

## Abstract

**Background:**

Hospital homepages should provide comprehensive information on the hospital's services, such as departments and treatments available, prices, waiting time, leisure facilities, and other information important for patients and their relatives. Norway, with its population of approximately 4.3 million, ranks among the top countries globally for its ability to absorb and use technology. It is unclear to what degree Norwegian hospitals and patients use the Internet for information about health services.

**Objectives:**

This study was undertaken to evaluate the quality of the biggest Norwegian cancer hospitals' Web sites and to gather some preliminary data on patients' use of the Internet.

**Methods:**

In January 2001, we analyzed Web sites of 5 of the 7 biggest Norwegian hospitals treating cancer patients using a scoring system. The scoring instrument was based on recommendations developed by the Norwegian Central Information Service for Web sites and reflects the scope and depth of service information offered on hospital Web pages. In addition, 31 cancer patients visiting one hospital-based medical oncologist were surveyed about their use of the Internet.

**Results:**

Of the 7 hospitals, 5 had a Web site. The Web sites differed markedly in quality. Types of information included - and number of Web sites that included each type of information - were, for example: search option, 1; interpreter service, 2; date of last update, 2; postal address, phone number, and e-mail service, 3; information in English, 2. None of the Web sites included information on waiting time or prices. Of the 31 patients surveyed, 12 had personal experience using the Internet and 4 had searched for medical information. The Internet users were significantly younger (mean age 47.8 years, range 28.4-66.8 years) than the nonusers (mean age 61.8 years, range 33.1-90.0 years) ( *P*= 0.007).

**Conclusions:**

The hospitals' Web sites offer cancer patients and relatives useful information, but the Web sites were not impressive.

## Introduction

Several investigators have shown that cancer patients consider information to be of great importance; further, informing patients and relatives is now an important part of cancer treatment [1,2]. During the last decade, information requests to health care workers in the Western world have steadily increased. Patients want to know more about the diagnosis, treatment, and follow-up of cancer. Questions about clinical results and hospitals' expertise are especially common. Many people have access to the Internet; in a Norwegian study [3], 63% had access to the Internet and 42% had their own PC with Internet access. The Internet has opened a new area to patients and their relatives. Much medical information is available directly at their homes at any time. But, even using the Internet, patients and relatives have difficulty finding the information they need. Excellent medical information exists, but it is scattered across dozens of different Web sites. The World Wide Web is the "wild wild Web." There is no comprehensive Web site that provides links to all the best online information for the patient's disease - this may be one of the major causes of the demand for more information and help from health professionals.

On January 1, 2001, the Norwegian National Health Administration introduced a new system named "free hospital selection" [4]. Until then, Norwegian patients had to be admitted to their local hospital according to geographic regulations. Based on the new legislation, cancer patients are now free to select among the different national public cancer hospitals. Only the increased travelling costs, if any, have to be paid by the individual patient. However, the National Insurance Scheme has decided to cover all travelling costs above a limit of US $44 (approximately Euro 50). As a result, patients can act as customers, buying the most attractive treatment. The different cancer institutions are put in competition to be attractive to the cancer patients. In this situation the hospital Web sites may be crucial because they may perform the same function that display windows do for stores.

To clarify whether the World Wide Web is likely to be an important platform for hospitals to advertise their services, one week after the introduction of "free hospital selection" a study on hospital Web sites was performed and a selected group of cancer patients were asked about their use of the Internet.

## Methods

### Web site evaluation

In January 2001, we looked for Web sites for the biggest Norwegian cancer hospitals. We found Web sites for these hospitals: The Norwegian Radium Hospital (NRH), Ullevål hospital, Haukeland University Hospital (HUH), Central Hospital of Rogaland (CHR), University Hospital of Trondheim (UHT), University Hospital of Tromsø and the Rikshospitalet University Hospital (RUH). These hospitals (except RUH) offered cancer patients radiotherapy, chemotherapy, hormonal therapy, and palliative therapy. When we could not find a Web site, we made a phone call to the hospital to confirm that there was no Web site. We found Web sites for the following hospitals: NRH (http://www.dnr.uio.no, now available at http://www.dnr.org), HUH (http://www.haukeland.no), CHR (http://www.sir.no), UHT (http://www.rit.no), and RUH (http://www.rikshospitalet.no). We analyzed the Web sites according to the scheme shown in Table 1. The scheme was based on Norwegian recommendations for Web sites developed by the Norwegian Central Information Service [5]. We gave a 0 to 3 score (0 = no information, 1 = a little information, 2 = some information, 3 = much information) to 7 items (items 1-4, 8-10). We gave a 0 to 1 score (0 = no information, 1 = information is given) to 6 items (items 5-7, 11-13). The maximum total score was 27 points. One rater employing a checklist performed all the ratings. The rater had no connection to any of the rated hospitals.

**Table 1:** Scheme Employed to Score the Information on the Hospital Web Pages

1. General information	(0-3 score)	Maps (of the area and the hospital), general description (location, taxi, bus, train) and information about car-parking
2. Addresses	(0-3 score)	Postal address, phone number, and e-mail address
3. Cancer department(s)	(0-3 score)	etc.)
4. Treatment available	(0-3 score)	institution
5. Price list	(0-1 score)	No price list = 0, any price list = 1
6. Search option	(0-1 score)	No search option = 0, any search option = 1
7. Interpreter service	(0-1 score)	No service offered = 0, any interpreter service = 1
8. Leisure facilities	(0-3 score)	For example: physical activities, library, bedside phone, Internet access, sightseeing, hairdresser
9. Links to databases	(0-3 score)	For example: The Norwegian Cancer Union, different medical journals
10. Relatives	(0-3 score)	and associated costs. Restaurant availability
11. Waiting time	(0-1 score)	No information = 0, any information = 1
12. Date of update	(0-1 score)	No date = 0, any date of update = 1
13. English version	(0-1 score)	No English version = 0, any English version = 1

### Patient survey

To get an idea of the use of the Internet by Norwegian cancer patients, 31 consecutive patients visiting one medical oncologist were interviewed. There were 21 women and 10 men; the majority of the patients suffered from breast cancer (15 patients), lymphoma (6 patients), or colorectal cancer (3 patients). Mean age was 56.3 years (range, 28.4-90.0 years). The interview was performed by the oncologist that the patients were visiting and took place at the outpatient clinic at the Department of Oncology, University Hospital of Tromsø (Tromsoe, Tromsö). During the outpatient visit, each patient was asked about any personal experience with the use of the Internet. If the patient responded positively, the interviewer asked whether the patient had used the Internet to gain access to medical information.

### Statistics

We used Microsoft Excel 97 for the final database and the Statistical Package for Social Science (SPSS) version 9.0 for statistical calculations. We used 1-way analysis of variance (ANOVA) to analyze for significant correlations. All *P* values are 2-tailed and considered statistically significant when *P*< 0.05.

## Results

### Web site evaluation

Of the 7 hospitals, 5 had a Web site on the Internet; the other 2 hospitals had plans for a running Web site within 2 months. We easily accessed the 5 Web sites using Microsoft Internet Explorer 3.0. The point scores for the Web sites were: Norwegian Radium Hospital, 15; Haukeland University Hospital, 10; Central Hospital of Rogaland, 6; University Hospital of Trondheim, 14; and the Rikshospitalet University Hospital, 13. Details on point scores are shown in Table 2.

**Table 2:** Scores of Hospital Web Sites

**Item**	**NRH**^a^	**HUH**^b^	**CHR**^c^	**UHT**^d^	**RUH**^e^	**Maximum score**
1. General information	2	2	1	3	2	3
2. Addresses	3	3	1	3	2	3
3. Cancer departments	1	1	0	1	1	3
4. Treatments available	2	2	0	2	2	3
5. Prices	0	0	0	0	0	1
6. Search option	0	0	0	1	0	1
7. Interpreter service	1	0	0	0	1	13
8. Leisure activities	3	1	1	0	2	3
9. Links	2	0	1	1	1	3
10. Relatives	0	1	1	2	1	3
11. Waiting	0	0	0	0	0	1
12. Date of update	1	0	1	0	0	1
13. English version	0	0	0	1	1	1
**Sum**	**15**	**10**	**6**	**14**	**13**	**27**

^a^ NRH = Norwegian Radium Hospital

^b^ HUH = Haukeland University Hospital

^c^ CHR = Central Hospital of Rogaland

^d^ UHT = University Hospital of Trondheim

^e^ RUH = National Hospital of Norway

Information about price lists or waiting time was not included on any of the Web sites. A search option was included on 1 Web site (UHT). Information on an interpreter service was included on 2 Web sites (NRH and RUH). The date of last update was included on 2 Web sites (NRH, CHR); the time since last update was 3.8 and 19.5 months, respectively.

The best general information was on the UHT Web site (this Web site received 3 points). This Web site included: a map of the area, an overview of the institution, details about car parking, and written information about how to reach the hospital by plane, train, bus and/or taxi.

Information - e-mail address, phone numbers, and postal address - on contacting the hospital was easily available on 3 Web sites., None of the 3 included e-mail addresses for either the departments or the oncologists, although all 3 had a central e-mail system. However, it was possible to find some direct e-mail addresses for the oncologists at the UHT by using the link - on the UHT Web site - to the University of Trondheim (http://www.ntnu.no). Information about the e-mail system - and about laws, regulations and risks related to mailing sensitive information - was included on the NRH Web site. The capability to search the hospital phone book by name, position, and department was included on the RUH Web site.

Information about hospital departments was very limited and was usually written; there were few pictures and no maps. Information on the treatments offered included only high-level summaries such as "radiotherapy, chemotherapy and hormonal therapy is offered." There were neither pictures nor illustrations. There were no details about the different treatments. The written information about hyperthermia at the HUH may act as a model for hospitals that want to improve the way they include treatment information on their Web sites.

The best leisure facilities information was on the NRH Web site (this Web site received 3 points). This Web site included information about such services as: cafeteria, kiosk, post office, bank, pharmacy, hairdresser, wig maker, makeup course, pedicure, hospital school, library, video, personal computers with games installed, bedside phone, television in all rooms, living room with a piano and a CD player, swimming pool, sauna, and exercise rooms. Information was included about possibilities for painting, carpentering, and sewing. The clergy offered devotions and services. Sightseeing tours and visits to museums, theaters, cinemas and football games as well as bicycles and cars were offered free of charge. Billiards, tennis and golf were also mentioned.

There was very limited information to help relatives on the Web sites. The best information to help relatives was on the UHT Web site (this Web site received 2 points). The UHT had made arrangements with 9 local hotels; hotel information included names, addresses and prices (590-829 Norwegian kroner/night, approximately 74-103 Euro/night). Some information about the cafeteria was included on the CHR and RUH Web sites. Some information about the possibility of staying at the hospital hotel was included on the HUH Web site.

Information in other language(s) was only on the UHT and the RUH Web sites. The UHT Web site included a summary in English and some information in German. The RUH Web site included information in English on treatment, teaching, staff, research, and development

### Patient survey

Only 12 out of 31 patients reported that they had any personal experience using the Internet. Of the 12, 4 (13% of the 31 patients surveyed) had searched for medical information on the Internet. The Internet users were significantly younger (mean age 47.8 years, range 28.4-66.8 years) than the nonusers (mean age 61.8 years, range 33.1-90.0 years) ( *P*= 0.007). We did not observe any difference in Internet use based on gender, type of cancer, or stage of disease (localized versus metastatic disease). However, the statistical power to detect differences in this pilot study was too low to make any reliable statements on lack of association between these variables and Internet use.

## Discussion

### Web site evaluation

This study has documented that only 5 of 7 major Norwegian hospitals had a running Internet Web site in January 2001. The quality of these Web sites differed markedly; score range was from 6 to 15 points. There was no information about price lists or waiting time, only limited information related to the departments and search options, and limited English summaries. However, some hospitals had very nice presentations about general information, ways to contact the hospital, and leisure facilities.

Price lists for treatment may have been omitted because all costs resulting from hospitalization are covered by the national public insurance, National Insurance Scheme (NIS). However, when patients are treated as outpatients, the patients and the NIS share the costs. Patients pay the same amount in all public hospitals and the hospitals are not allowed to make special offers.

Since the 5 institutions are research centers taking part in national and international research projects, it is disappointing that only 2 institutions offered an English summary. Factors that may require English information on the hospitals' Web sites include tourism, immigration, and patients from foreign countries seeking medical treatment or advice in Norway.

In this study, 3 of the 5 Web sites provided e-mail interactivity. This percentage (60%) is somewhat lower than the finding of Hoffman-Goetz and Clarke that 88% of the breast cancer sites on the World Wide Web provided this service [6]. It is generally recommended that Web sites provide a method for users to correct wrong information and report failures. There are reasons to believe that in the future patients will want to communicate with doctors directly instead of through a hospital's central e-mail system. This statement is based on the experience that Norwegian cancer patients consider their oncologist to be the most important source of information about the disease (Norwegian Centre for Telemedicine, oral communication, December 2001) and on individual patient-doctor relations created during visits at the outpatient clinics. Although this direct communication is technically possible in Norway, there are several security concerns that have to be solved, because connecting PCs both to the Internet and to a hospital intranet containing patient and hospital data may make it possible to manipulate that data from the Internet.

### Patient survey

We found a significant correlation between patients' age and the use of Internet. This is in accordance with a Norwegian survey [3] that documented a correlation between age below 60 years and experience with the Internet. The Norwegian survey also observed a difference based on gender, as males were more frequently Internet users. Level of education may be another factor in Internet use. Other investigators have documented that patients with longer formal education have a more active information-seeking strategy than those with a more limited formal education [7-9].

### Conclusions

Knowing that there will be increased competition between the hospitals, since Norwegian patients are now offered the possibility of selecting their hospital for treatment, and assuming that hospital Web sites may perform the function for patients selecting their hospital that display windows perform for stores, the Web sites were not impressive.

Our finding that few cancer patients (13%) had sought medical information on the Internet is comparable to other surveys. The results have to be interpreted with caution because this study lacks statistical power and does not use a large cross section of patients. However, the figures are in accordance with the results from a Swedish study done by Carlsson in Uppsala finding that only 6% of adult patients visiting the Department of Oncology had sought information from the Internet [2]. Another study performed by the Norwegian Centre for Telemedicine (NCT) documented that 19% of the Norwegian population had employed the Internet to gain access to medical information [10]. These results are surprising, particularly because Scandinavian countries have one of the highest Internet penetrations in the world.

It could be argued that there is no need to allocate resources to the development of Web sites, because only a few patients search for medical information on the Internet. However, there are several reasons to believe that this will change as more and more people gain access to the Internet. It has been estimated that about 500 million computers were linked to the Internet at the end of the year 2000 [11]. There are reasons to believe that in the future Intranets and the Internet will be more important in informing and communicating with cancer patients and their relatives.

## Abbreviations

CHR: Central Hospital of Rogaland
HUH: Haukeland University Hospital
NIS: National Insurance Scheme
NRH: Norwegian Radium Hospital
RUH: National Hospital of Norway
UHT: University Hospital of Trondheim
